# Left internal iliac artery stenosis as a risk factor for anastomotic leakage after left-sided colorectal surgery

**DOI:** 10.3389/fsurg.2026.1770783

**Published:** 2026-05-07

**Authors:** Sonaz Malekzadeh, Salah Dine Qanadli, Joana Ribeiro Mateus, Leonid Tsepenshchikov, Ian Fournier, Ioannis Rotas, Boumediene Guendil, Daniel Clerc, Lucien Widmer

**Affiliations:** 1 Department of Diagnostic and Interventional Radiology, Hôpital du Valais, Sion, Switzerland; 2 Faculty of Biology and Medicine, University of Lausanne, Lausanne, Switzerland; 3 Clinical Research Unit, Riviera-Chablais Hospital, Rennaz, Switzerland; 4 Department of Surgery, Hôpital du Valais, Sion, Switzerland; 5 Department of Medical and Surgical Specialties, Faculty of Science and Medicine, University of Fribourg, Fribourg, Switzerland; 6 Department of Radiology, University Training and Research Hospital of Fribourg (HFR), Fribourg, Switzerland

**Keywords:** anastomotic leakage (AL), arterial stenosis, computed tomography, left-sided colorectal resection, predictor

## Abstract

**Purpose:**

To evaluate association of stenosis of celio-mesenteric and internal iliac arteries with anastomotic leakage (AL) following left-sided colorectal resection in patients with primary colorectal cancer.

**Methods:**

This single-center study included consecutive patients undergoing left-sided colorectal resection for primary colorectal cancer between 2014 and 2024. Patients with and without AL were matched by propensity scores. Two radiologists independently assessed preoperative computed tomography (CT) scans for arterial stenosis (<50% vs. ≥50%). Weighted multivariate logistic regression identified independent predictors of leakage.

**Results:**

Among 367 consecutive left-sided colorectal resections, 37 patient with AL were eligible and matched to 60 controls for detailed vascular analysis. Baseline characteristics were well balanced after matching. Vascular assessment on CT showed a prevalence of significant stenosis ranging from 9.3% to 23.7%, with good to excellent interrater agreement (*κ*≥0.71, *p* < 0.001). Propensity matching achieved covariate balance for most of variables (SMDs <0.1). Weighted multivariate regression identified left internal iliac artery (LII) stenosis as significantly associated with AL (OR 4.13, *p* = 0.026). No association was observed between AL and the celiac artery, superior mesenteric artery, inferior mesenteric artery, or right internal iliac.

**Conclusion:**

LII stenosis emerged as the most relevant vascular predictor of AL after left-sided colorectal surgery. Incorporating preoperative arterial assessment on routine contrast-enhanced CT may therefore improve risk stratification and guide preventive strategies in selected patients.

**Statement:**

This study identifies left internal iliac artery stenosis as an independent vascular predictor of anastomotic leakage after left-sided colorectal resection. Incorporating preoperative arterial assessment into routine CT may therefore improve risk stratification and guide preventive strategies in selected patients.

## Introduction

Colorectal cancer is the third most frequent cancer around the world, and curative intent surgery remains the primary therapeutic approach to be adopted when indicated (1, 2). Anastomotic leakage (AL) represents one of the most serious postoperative complications, occurring in between 1.5% and 20% of patients (3). AL is associated with prolonged hospital stay, increased morbidity and mortality, as well as increased risk of local oncological disease recurrence (4). These risks highlight the clinical imperative for precise preoperative risk assessment to guide selective protective stoma creation.

Multiple factors have been demonstrated to be involved in the development of AL, encompassing patient-related, perioperative considerations, and operation technique. These include age, sex, smoking, body mass index (BMI), American Society of Anesthesiologists (ASA) score, nutritional status, anastomotic height, intraoperative bleeding, neoadjuvant radiotherapy, stapled vs. hand-sewn anastomosis, standard laparoscopy vs. open surgery, as well as surgeon experience (5–14). Despite significant improvements in modifiable factors, including nutritional optimization, perioperative care, surgical technique, and surgeon experience, the rate of AL remains significant. This suggests the possibility of other factors contributing to AL.

Atherosclerosis has been demonstrated to cause local perfusion alteration and subsequently altered tissue healing by restricting blood flow. However, previous studies have produced inconsistent results regarding the role of ischemia in anastomotic healing following colorectal resection. While some studies have shown a correlation between atherosclerosis and AL due to inadequate anastomotic perfusion, other have demonstrate no such association (15–17). The inferior mesenteric artery (IMA) plays the pivotal role in the vascularization of the left-sided colon, upper and middle rectum. In contrast, the lower rectum is mainly irrigated by branches of the internal iliac arteries (18, 19). Additionally, the collateral network enables the celiac artery (CA) and superior mesenteric artery (SMA) to provide supplementary perfusion to the vascular territory of the IMA. In patients with colorectal cancer, computed tomography (CT) not only provides crucial information on oncological staging, but also offers important insights into vascular assessment.

This study aims to demonstrate the effect of stenosis of CA, SMA, IMA, right internal iliac artery (RII), and left internal iliac artery (LII) on AL following left-sided colorectal resection in patients with primary colorectal cancer.

## Method

This was a retrospective single-center observational cohort study conducted at a cantonal referral hospital. All eligible patients were identified from the institutional Enhanced Recovery after Surgery (ERAS) registry, which prospectively record perioperative and outcome data for consecutive colorectal procedures. They were crosschecked with radiology and surgical reports to ensure complete capture of AL events. The institutional review board approved the study and all patients provided informed consent. The design, conduct and reporting of this study were aligned with the STROBE (Strengthening the reporting of observational studies in epidemiology) recommendations for observational studies.

### Study population

**Figure 1:**
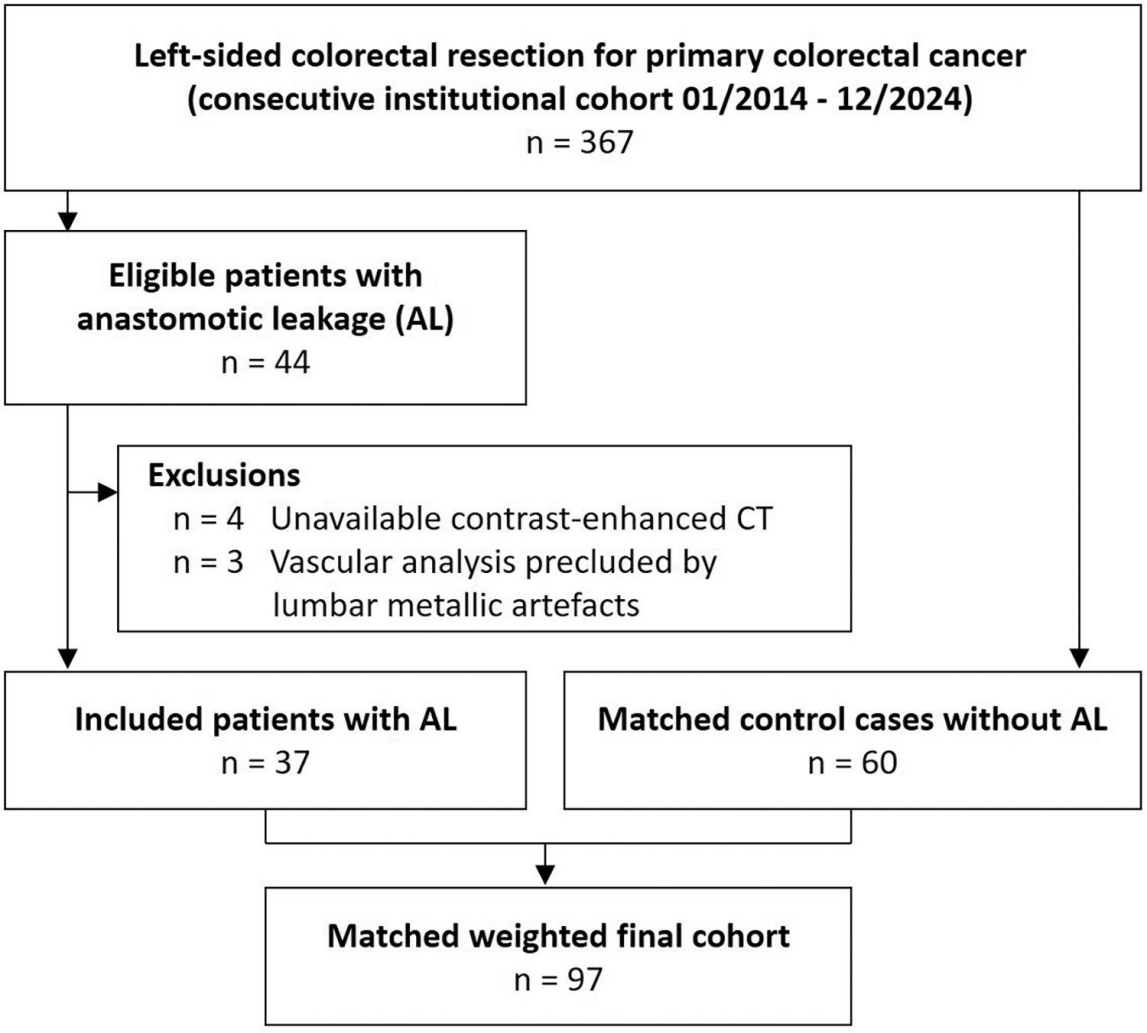
Flowchart of the study.

All consecutive patients who underwent left-sided colorectal resection for primary tumors with direct anastomosis, without formation of a protective stoma, between January 2014 and December 2024 were screened for eligibility. All patients had undergone a contrast-enhanced abdominal CT scan within one month prior to surgery. Patients who developed AL within 90 days post-operatively were classified as cases, and those without evidence of AL during the same period were eligible as controls. Exclusion criteria were previous neoadjuvant therapy, previous history of colon resection or abdominal revascularization, left-sided colon resection due to secondary tumoral involvement, incomplete clinical data, unavailable contrast-enhanced CT, or vascular analysis precluded by lumbar metallic beam hardening artefacts. To control for confounding, patients without evidence of AL were frequency matched from the same registry. The number of patients excluded for each criterion, as well as the final numbers of included cases and controls, are detailed in Figure 1.

### Clinical data

Medical records were reviewed to extract demographic variables, comorbidities, and perioperative data. Variables included gender, age, BMI, hypertension (HTN), diabetes mellitus (DM), and smoking status. ASA score was retrieved from anaesthesiology charts, with patients with ASA grade ≥ III considered at higher risk of post-operative complication development (20). Nutritional status was evaluated by the Nutritional Risk Screening (NRS) score, with patients with grade ≥3 considered to be at risk for malnutrition (21). Surgical procedures comprised left colectomy, sigmoid resection, high anterior resection, and low anterior resection. Urgency of surgery (elective vs. urgent) and surgical approach (standard laparoscopy vs. open surgery) were also collected.

### Post-operative complications

AL was defined as a loss of integrity at the colorectal anastomotic site, resulting in a communication between the intraluminal and extraluminal compartments (22). The diagnosis of AL was established based on findings from CT, through surgical drain, or intraoperative confirmation during subsequent reoperation. ALs were further divided into three groups (group A, B or C) according to the adopted treatment strategy: patients with grade A leaks were those managed by conservative treatment, while grades B and C leaks where those requiring radiological and surgical intervention, respectively (23).

### Imaging analysis

All CT examinations were independently reviewed by two radiologists, with 8- and 4-years' experience in abdominal imaging, respectively. Inter-rater agreement was quantified using Cohen's Kappa statistic. Scans were acquired on various CT platforms, as some patients were referred from external institutions. For all patients, portal venous phase abdominal CT with a slice thickness of ≤3 mm and access to multiplanar reformats (MPR) were available. CA, SMA, IMA, LII, and RII were analysed, and the degree of stenosis was classified into as either <50% or ≥50%. The percentage of stenosis was calculated as the ratio of the narrowest luminal diameter to the widest luminal diameter distal to the stenosis. Each vessel was assessed on at least two different orthogonal planes (axial, coronal, and sagittal) to reduce the effect of partial volume. Discrepancies in SMA assessment were resolved by consensus.

## Statistical analysis

### Matching procedure

To reduce confounding and improve the covariate balance between patients with and without anastomotic leakage, we implemented propensity score matching using full matching approach via MatchIt package in R (version 4.5.1). The propensity score was estimated with a logistic regression model including gender, age, BMI, HTN, DM, smoking status, ASA score, NRS, urgency of surgery and surgical approach. Full matching was then applied to create weighted matched sets that included all cases of AL and available controls from the consecutive institutional cohort. This approach assigns weights to individuals according to the quality of their matches, thereby constructing a weighted pseudo-population in which the distribution of covariates is more similar between groups. Covariate balance was assessed using standardized mean differences (SMDs), with values <0.1 considered indicative of adequate balance. When residual imbalances persisted (SMD ≥0.1), the corresponding variables were additionally included as covariates in the multivariable logistic regression models to further mitigate confounding.

### Univariate analyses

After matching, weighted univariate logistic regression models were fitted with AL as the dependent variable and each candidate predictor as the independent variable. Predictors included demographics (gender, age, BMI, smoking status), comorbidities (HTN, DM), perioperative factors (ASA score, NRS score, surgical urgency and surgical approach), and arterial stenosis variables (CA, SMA, IMA, RII, and LII stenosis).

### Multivariate analyses

Multivariable logistic regression models were constructed to evaluate the association between each arterial stenosis and AL in the matched pseudo-population. Given the limited number of leakage events, we developed separate models for each arterial stenosis variable (CA, SMA, IMA, RII, and LII). For each artery, the model included that stenosis variable together with clinically relevant confounders and covariates that had not already been balanced by matching, thus intentionally being restricted to preserve parsimony and avoid overfitting. Univariate analyses were used descriptively and did not determine model inclusion.

### Model validation and robustness

To evaluate the reliability of the multivariate logistic regression models, three diagnostic and validation procedures were conducted. Multicollinearity among covariates was assessed using variance inflation factors (VIFs), with values <2 considered acceptable. Model calibration was examined using the Hosmer–Lemeshow goodness of fit test with grouped deciles of risk; non-significant *p* values (*p* > 0.05) were interpreted as evidence of adequate calibration. Discrimination was quantified by the c statistic (AUC), with AUC ≥0.6 considered fair, ≥0.7 acceptable, ≥0.8 good, and ≥0.9 excellent.

Categorical variables were reported as absolute numbers and percentages, and continuous variables were presented as mean ± standard deviation. Results from univariate and multivariate analyses and models were reported as odds ratios (OR) with their corresponding 95% confidence intervals (CI) to facilitate interpretation of effect sizes. Confidence intervals were derived from the regression coefficients using the formula: β ± 1.96 × SE.

## Results

A total of 367 procedures were reviewed, with 44 AL events reported (incidence 11.9%). Seven patients with AL were excluded according to predefined exclusion criteria. The remaining 37 patients with AL were matched with 60 controls. The matched analytic sample comprised 37 AL cases and 60 controls (*n* = 97), included 54 men (55.7%), with a mean age of 67 ± 12 years and BMI 26.5 ± 5 kg/m^2^. Thirty-eight patients (39.2%) had HTN, and 17 (17.5%) had DM. Fifteen patients (15.5%) were active smokers. Most surgeries were elective (79, 81.4%), and 61 (62.9%) were laparoscopic. Among patients with AL, 32 (86.4%) required reoperation. Baseline characteristics of study population are detailed in Table 1.

**Table 1:** Baseline demographic and clinical characteristics of patients with and without anastomotic leakage.

Variable	Anastomotic leakage (*n* = 37)	No leakage (*n* = 60)	Total (*n* = 97)	*p* value
Gender, *n* (%)	Male 22 (59.5)	32 (53.3)	54 (55.7)	0.555
Age, years (mean ± SD)	69.8 ± 12.2	65.7 ± 11.9	67.0 ± 12.0	0.111
BMI, kg/m^2^ (mean ± SD)	27.0 ± 4.8	26.1 ± 5.2	26.5 ± 5.0	0.390
Hypertension, *n* (%)	18 (48.6)	20 (33.3)	38 (39.2)	0.133
Diabetes mellitus, *n* (%)	10 (27.0)	7 (11.7)	17 (17.5)	0.053
Smoking status, *n* (%)	Active 6 (16.2)	9 (15.0)	15 (15.5)	0.952
	Ex-smoker 10 (27.0)	15 (25.0)	25 (25.8)	
	Non-smoker 21 (56.8)	36 (60.0)	57 (58.8)	
ASA score ≥ III	22 (59.5)	24 (40.0)	46 (47.4)	0.062
NRS score ≥3	25 (67.6)	36 (60.0)	61 (62.9)	0.454
Surgical urgency, *n* (%)	Elective 33 (89.2)	46 (76.7)	79 (81.4)	0.123
	Urgent 4 (10.8)	14 (23.3)	18 (18.6)	
Surgical approach, *n* (%)	Open surgery 16 (43.2)	20 (33.3)	36 (37.1)	0.326
	Laparoscopy 21 (56.8)	40 (66.7)	61 (62.9)	
Leakage treatment strategy, *n* (%)	Surgery 32 (86.4)			
	Percutaneous drainage 3 (8.1)			
	Conservative 2 (5.4)			

Values are presented as mean ± standard deviation (SD) for continuous variables and as number (percentage) for categorical variables. *P* values are derived from independent samples *t* tests for continuous variables and Chi square or Fisher’s exact tests for categorical variables, comparing leakage vs. non-leakage groups. BMI, body mass index; ASA, American Society of Anesthesiologists; NRS, Nutritional Risk Screening.

Table 2 presents stenosis prevalence across the five arteries studied, stenosis evaluation by each rater and inter-rater agreement for stenosis assessment. Inter-rater agreement was perfect for RII stenosis (*κ* = 1.00, *p* < 0.001), very good for CA, IMA and LII stenoses (*κ* = 0.97, 0.97 and 0.93, respectively, with *p* < 0.001 for all), and good for SMA stenosis (*κ* = 0.708, *p* < 0.001). Given the overall high concordance, the evaluation of one radiologist was used for the primary analyses, with consensus ratings applied in cases of disagreement.

**Table 2:** Significant stenosis prevalence across arteries and inter-rater agreement for stenosis assessment.

Artery	Rater 1	Rater 2	Kappa value (CI 95%)	*p* value
Celiac	22.7%	23.7%	0.97 (0.91–1.0)	<0.001
SMA	9.3%	10.3%	0.708 (0.47–0.95)	<0.001
IMA	22.7%	23.7%	0.97 (0.91–1.0)	<0.001
LII	18.5%	18.5%	0.93 (0.84–1.0)	<0.001
RII	16.5%	16.5%	1 (1.0–1.0)	<0.001

Significant arterial stenosis was defined as a reduction of ≥50% of artery diameter. Kappa value < 0.20: Poor agreement, 0.21–0.40: Fair agreement, 0.41–0.60: Moderate agreement, 0.61–0.80: Good agreement, 0.81–1.00: Very good agreement. SMA, superior mesenteric artery; IMA, inferior mesenteric artery; LII, left internal iliac artery; RII, right internal iliac artery.

### Matching

Full matching retained all 37 AL patients and 60 controls. After weighting, the effective control sample size corresponded to 21.5 subjects. Post-matching SMDs were below 0.1 for surgical approach, urgency, ASA, gender, DM, and tobacco use, indicating acceptable covariate balance. NRS score showed a borderline imbalance (SMD = -0.113), while hypertension, BMI, and age remained more imbalanced with post-matching SMDs of –0.200 and 0.146, and 0.313, respectively. The overall propensity score distance decreased from 0.767 before matching to 0.161 after matching, reflecting improved comparability between groups. Residual imbalance for age and HTN after matching was addressed by including these variables in subsequent multivariable regression models.

### Univariate analyses

In weighted univariate logistic regression, the following factors were not associated with leakage (*p* ≥ 0.10): gender, ASA, NRS, BMI, DM, smoking status, surgical approach, surgical urgency, and IMA stenosis (*p* = 0.144). Borderline associations (0.05 ≤ *p* < 0.10) was observed with age (0.09), and HTN (*p* = 0.05). Significant predictors (*p* < 0.05) were demonstrated to be Celiac stenosis (*p* = 0.023), SMA stenosis (*p* = 0.034), RII stenosis (*p* = 0.044), and LII stenosis (*p* = 0.015). Table 3 shows the univariate logistic regression for predictors of AL.

**Table 3:** Univariate logistic regression for predictors of anastomotic leakage (after full matching).

Variable	OR [Exp(B)]	95% CI	*p* value	Inclusion in multivariate analysis
Gender	0.86	0.37–2.02	0.734	No
Age	1.03	0.99–1.08	0.099	**Yes**
BMI	1.04	0.94–1.14	0.450	No
Hypertension	0.42	0.18–0.99	0.049	**Yes**
Diabetes mellitus	1.37	0.56–3.46	0.498	No
Smoking status	0.76	0.24–2.16	0.612	No
ASA	1.05	0.46–2.43	0.913	No
NRS	0.56	0.22–1.42	0.220	No
Surgical approach	0.73	0.31–1.69	0.455	No
Surgical urgency	0.72	0.18–2.42	0.606	No
Celiac stenosis	2.99	1.17–7.90	0.023	**Yes**
SMA stenosis	4.85	1.22–25.3	0.034	**Yes**
IMA stenosis	2.14	0.77–6.03	0.144	**Yes**
LII stenosis	4.34	1.38–15.6	0.015	**Yes**
RII stenosis	3.32	1.06–11.4	0.044	**Yes**

Bold values indicate inclusion of the variable in the multivariate analysis.

Odds ratios (OR) are calculated as exp(estimate). 95% confidence intervals (CI) are approximate, based on ± 1.96 × SE. Arterial stenosis was defined as a reduction of ≥50% of artery diameter. BMI, body mass index; ASA, American Society of Anesthesiologists physical status classification system; NRS, Nutritional Risk Screening score; SMA, superior mesenteric artery; IMA, inferior mesenteric artery; LII, left internal iliac artery; RII, right internal iliac artery.

### Multivariate analyses

Multivariate logistic regression analyses were performed including age, HTN (the two clinically relevant variables that did not show satisfactory adjustment during matching procedure) and one arterial stenosis variable at a time. LII stenosis was significantly associated with anastomotic leakage, corresponding to a more than fourfold increase in odds (OR 4.13, 95% CI 1.24–15.7, *p* = 0.026). In this model, HTN (OR 0.44, 95% CI 0.17–1.11, *p* = 0.084) and age (OR 1.01, 95% CI 0.97–1.06, *p* = 0.568) were not significantly associated. No significant association was observed between AL and CA, SMA, IMA, or RII (*p* = 0.065, 0.087, 0.042, and 0.12, respectively). Table 4 summarizes the multivariate logistic regression analysis for AL predictors.

**Table 4:** Weighted multivariate logistic regression models for predictors of anastomotic leakage.

Model (artery)	Predictor	OR	95% CI	*p* value
Celiac	Hypertension	0.51	0.20–1.27	0.14
	Age	1.02	0.97–1.06	0.44
	Celiac	2.53	0.94–6.91	0.065
SMA	Hypertension	0.55	0.22–1.38	0.19
	Age	1.02	0.97–1.07	0.33
	SMA	3.69	0.88–19.8	0.087
IMA	Hypertension	0.53	0.21–1.34	0.18
	Age	1.02	0.98–1.07	0.29
	IMA	1.55	0.52–4.61	0.42
LII	Hypertension	0.44	0.17–1.11	0.08
	Age	1.01	0.97–1.06	0.56
	LII	4.13	1.24–15.7	**0**.**026**
RII	Hypertension	0.52	0.21–1.30	0.16
	Age	1.02	0.97–1.07	0.386
	RII	2.60	0.79–9.26	0.12

Bold values indicate significant association of predictor with anastomotic leakage.

Multivariate logistic regression models were constructed separately for each arterial stenosis (celiac, SMA, IMA, LII, RII), adjusted for hypertension, and age. Odds ratios (OR) with 95% confidence intervals (CI) are shown. LII stenosis was significantly associated with anastomotic leakage, while SMA stenosis showed a strong but non-significant trend. Other arteries and covariates were not significantly associated. SMA, superior mesenteric artery; IMA, inferior mesenteric artery; LII, left internal iliac artery; RII, right internal iliac artery.

Among the arterial territories evaluated, LII stenosis demonstrated the strongest association with AL in adjusted analyses, reaching statistical significance, while CA and SMA stenosis demonstrated borderline associations. HTN and age did not demonstrate significant associations across models.

### Model validation and robustness

For the LII stenosis multivariate logistic regression model, variance inflation factors (VIFs) for hypertension, age, and LII stenosis were all close to 1 (range 1.07–1.14), indicating no evidence of problematic multicollinearity. Model calibration was acceptable, as demonstrated by a non-significant Hosmer–Lemeshow goodness-of-fit test (*χ*^2^ = 6.57, df = 8, *p* = 0.584). Discrimination was modest, with an area under the ROC curve (AUC) of 0.65, reflecting limited ability to distinguish between patients with and without anastomotic leakage.

## Discussion

The present study identified a significant association between LII stenosis and AL in patients undergoing left-sided colorectal resection with primary anastomosis (*p* = 0.026). Multivariate analysis indicated more than fourfold increased risk of AL in patients with LII stenosis ≥50%. No significant associations were observed for CA, IMA, and RII stenoses.

Several demographic and perioperative factors such as age, sex, DM, HTN, NRS score, ASA score, surgical approach and urgency have been implicated in the development of AL (11, 24). Accurate preoperative risk assessment of AL could improve patient selection for preventive procedures. Despite advances in surgical techniques, surgeon experience, and improvements in perioperative management, the rate of AL remains considerable, suggesting that additional factors may play a critical role (3, 25). Adequate perfusion at the anastomotic site is essential for healing and structural integrity (26). Both experimental and clinical studies have demonstrated the impact of impaired microcirculation on the development of AL. In a rat model, Czeiger et al. showed that hypoperfusion impairs anastomotic strength in colon anastomosis (27). Using intraoperative laser Doppler in patients with rectal cancer, Vignali et al. demonstrated that the altered blood flow at the rectal stump is associated with increased AL rate (28). Although colorectal perfusion cannot typically be routinely quantified on preoperative imaging, pre-surgical contrast-enhanced CT allows indirect evaluation of vascular status through the assessment of major visceral and pelvic arteries.

Vascular obstruction on CT can be quantified using calcium scoring, but this method has notable limitations (29, 30). Firstly, it requires dedicated softwares that are not universally available. Secondarily, it has been demonstrated that in coronary artery nearly 20% of atherosclerosis plaques are calcified which could be detected and measured by calcium score program. However, nearly 80% of plaques are lipid-rich and fibrotic, which are undetectable by calcium score software and may therefore underestimate the actual plaque load in vessels (31, 32). On the other hand, by measuring the luminal vessel diameter, as in the present study, all types of plaques are evaluated, making this a more reliable technique. A luminal reduction of ≥50% is usually considered as a hemodynamically significant stenosis while ≥70% is defined as severe stenosis, acknowledging that these thresholds are primarily based on studies involving arterial territories other than the iliac arteries (33). In the present study, due to the limited number of patients with stenosis >70%, the threshold was based on the stenosis of ≥50%, consistent with prior comparative studies (15–17).

Previous research evaluating the relationship between arterial stenosis and AL has produced inconsistent results, partly due to methodological differences. In a large population-based matched study, Arron et al. evaluated the relationship between arterial stenosis of ≥70% and AL in patients who underwent left-sided colorectal resection (34). Among the CA, SMA, and IMA, only IMA stenosis showed a significant association with AL. The stricter stenosis threshold (≥70% vs. ≥50% in the present study) likely explains the discrepancy. Boersema et al., in matched groups of patients who underwent left-sided colectomy, found no association between the stenosis of iliac arteries and AL except for RII that they considered as a probable coincidence (35). However, they examined arterial stenosis using the calcium score rather than the luminal diameter assessment, which may affect the comparability of results with the present study. Using a similar threshold for arterial stenosis of ≥50%, Kornmann et al. found no significant association between stenosis of the CA, SMA, IMA, or iliac arteries and AL in a large cohort of patients underwent colorectal resection (15). However, design potentially allowed their unmatched confounding effects.

Anatomically, The IMA is the main arterial supply of the left colon, sigmoid, and upper rectum, while the LII predominantly vascularizes the mid and lower rectum. Variability of left colonic vascular anatomy and differences in marginal perfusion zones may increase the vulnerability of certain patients to critical ischemia despite only moderate degrees of stenosis. The use of three-dimensional vascular mapping based on CT angiography in elective cases could enhance recognition of vascular anatomy and potential variations, thereby improving surgical planning and potentially reducing ischemic risk (36). In our study, the significant association between LII stenosis and AL, but not with IMA stenosis, may reflect recent surgical advances. Techniques such as high vs. low ligation of the IMA and the use of indocyanine green (ICG) fluorescence perfusion imaging allow surgeons to obtain a well-perfused proximal resection margin (37, 38). These innovations likely mitigate the impact of IMA stenosis on anastomotic perfusion. However, they may also indicate that the distal segment of the anastomosis, mainly irrigated by LII, receives comparatively less attention, which could explain the persisting influence of LII insufficiency on AL risk.

Regarding the influence of vascular stenosis on AL, two main hypotheses have been proposed. Some authors believe that in patients with significant arterial stenosis due to the atherosclerosis, tissue vascularization may be sufficient under normal conditions, but following colorectal resection, this compromised arterial network may fail to ensure adequate perfusion, thereby predisposing to AL (17, 39). Conversely, some authors suggest the presence of significant arterial stenosis due to atherosclerosis promotes the development of rich collateral vascular network, which may act as the protective effect on colorectal anastomosis integrity while resection (15, 40). The findings of the present study support the first hypothesis, demonstrating that significant stenosis of LII and SMA is associated with increased risk of AL. In elective setting, preoperative endovascular intervention, such as balloon angioplasty with or without stenting, may therefore merit consideration for patients with LII or SMA stenosis of ≥50% undergoing left-sided colorectal resection. However, in emergency procedures for the same patient population, the formation of a diverting stoma may represent an alternative preventive strategy.

This study has several limitations that affect the interpretation and external validity of our findings. First, its retrospective design induces potential selection bias. In addition, the relatively small sample size limits the statistical power and constrained multivariable adjustment. This limited number of patients is partly explained by the inclusion criteria, as only individuals who underwent left-sided colon resection were considered. Because tumor location is known to influence the risk of AL, we restricted the study population to left-sided colorectal resections in order to minimize this confounding effect. Although we attempted to match some relevant variables between groups, it was not possible to control for all known risk factors due to the large number of variables and the limited sample size. Nevertheless, we included the most pertinent variables, such as surgical approach and urgency, which are likely to have a substantial impact on the outcomes. The limited number of leakage events may compromise the stability of the multivariable estimates. Although models were intentionally restricted to preserve parsimony and maintain an acceptable events-per-variable ratio, findings should be interpreted cautiously. The two-step strategy (propensity score full matching followed by multivariable adjustment) was chosen to reduce, but cannot fully exclude, residual confounding. The propensity score full matching created a weighted pseudo-population that improved internal comparability, but necessarily shifts the focus from the raw consecutive cohort to and adjusted matched sample. Thus, our results should be considered as hypothesis-generating, as the associations observed refer primarily to patients whose characteristics were represented in this matched pseudo-population. Therefore, extrapolation to broader colorectal surgical populations should be undertaken with caution, and external validation in larger cohorts is warranted. Another limitation is the use of different CT scanners and imaging protocols across patients. Variability in scanner type, acquisition parameters, and reconstruction techniques may have introduced heterogeneity in image quality, potentially affecting the assessment of arterial stenosis, especially in quantifying stenosis in small arteries (IMA). Although standardized criteria were applied by the radiologists, this technical variability could have influenced the consistency of stenosis classification and should be considered when interpreting the findings. In addition, the vascular analyses were performed using the portal phase acquisition, which may have influenced the results. However, because preoperative CT is routinely performed in the portal venous phase, this approach enhances the reproducibility of the results in daily clinical practice.

In conclusion, we observed an association between left internal iliac artery stenoses and anastomotic leakage after left-sided colorectal resection in a matched weighted cohort. Larger, multicenter prospective studies are warranted to confirm these exploratory findings and to define appropriate preoperative risk-stratification and intervention strategies. These strategies include the role of preoperative endovascular intervention of LII in elective patients or performing a diverting stoma in emergency settings.

## Data Availability

The raw data supporting the conclusions of this article will be made available by the authors, without undue reservation.
